# How to perform left and right ventricular function quantification in cardiac magnetic resonance imaging with a simple mouse click

**DOI:** 10.1186/1532-429X-13-S1-P139

**Published:** 2011-02-02

**Authors:** Lambert R Masip, Miguel Souto, Pablo G Tahoces, Amparo Martínez, Juan J Vidal

**Affiliations:** 1 University of Santiago de Compostela, Santiago de Compostela, Spain; 2 University Hospital Complex of Santiago de Compostela (CHUS), Santiago de Compostela, Spain

## Introduction

Ventricular function is a primary indicator for the diagnosis and treatment monitoring of many cardiovascular diseases. Cardiac cine magnetic resonance imaging (MRI) with steady state free precession (SSFP) sequences is regarded to be the standard of reference for the assessment of ventricular function. However, manual segmentation of MRI data is a time consuming process and also suffers from inter/intra-observer variability. This justifies the development of more automated segmentation methods to reduce the amount of time and effort that an experienced operator must spend on this process, and to make such methods practical.

## Purposes

The objective of this study was to validate our automated segmentation method developed to perform left and right ventricular function quantification from cardiac magnetic resonance images with a simple mouse click.

## Methods

**Figure 1:**
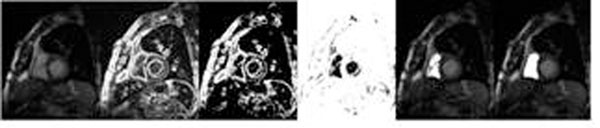
Automated segmentation scheme: User interaction was reduced to a mouse click on the analyzed ventricle in a midventricular ED frame. Then, a square kernel was set up around the mouse click position and all the frames were automatically segmented.

Thirty patients with cardiovascular diseases were examined using a 1.5-T MRI unit (Magnetom Symphony Maestro Class, syngo MR 2002B software; Siemen AG, Medical Solutions, Erlangen, Germany). A stack of short axis slices with a thickness of 6-mm was planned according to the vertical and horizontal long-axis views, to cover both ventricles from the base to the apex. Acquisition was performed with end-expiratory breath-hold cine SSFP sequences. Several parameters such as end-diastolic and end-systolic volumes (EDV and ESV, respectively), stroke volume (SV), and ejection fraction (EF) of both ventricles were quantified using our automated segmentation method based on edge detection, iterative thresholding and region growing techniques (Figure 1). Obtained results were validated against those performed using a commercially available software package based on manual contour tracing (Argus, release syngo MR 2002B; Siemens AG, Medical Solutions, Erlangen, Germany).

## Results

No statistically significant differences were found for the quantification of left and right ventricular (LV and RV, respectively) parameters using both methods (p>0.05). Correlation to estimate RV function (r>0.7) was slightly lower than that obtained for the left ventricle (r>0.9). Bland-Altman plots were used to assess the agreement between both methods.

## Conclusions

In conclusion, our automated segmentation method: provides similar results to those achieved by manual contour tracing, reduces both the time employed and the inter/intra-observer variability to a mouse click, and does not rely on a prior knowledge, providing a true segmentation of the anatomical features present in the image.

